# Duodenal Lipid Sensing Activates Vagal Afferents to Regulate Non-Shivering Brown Fat Thermogenesis in Rats

**DOI:** 10.1371/journal.pone.0051898

**Published:** 2012-12-14

**Authors:** Clémence Blouet, Gary J. Schwartz

**Affiliations:** Departments of Medicine and Neuroscience, Diabetes Research Center, Albert Einstein College of Medicine, Bronx, New York, United States of America; Pennington Biomed Research Center, United States of America

## Abstract

Previous evidence indicates that duodenal lipid sensing engages gut-brain neurocircuits to determine food intake and hepatic glucose production, but a potential role for gut-brain communication in the control of energy expenditure remains to be determined. Here, we tested the hypothesis that duodenal lipid sensing activates a gut–brain–brown adipose tissue neuraxis to regulate thermogenesis. We demonstrate that direct administration of lipids into the duodenum increases brown fat temperature. Co-infusion of the local anesthetic tetracaine with duodenal lipids abolished the lipid-induced increase in brown fat temperature. Systemic administration of the CCKA receptor antagonist devazepide blocked the ability of duodenal lipids to increase brown fat thermogenesis. Parenchymal administration of the N-methyl-d-aspartate receptor blocker MK-801 directly into the caudomedial nucleus of the solitary tract also abolished duodenal lipid-induced activation of brown fat thermogenesis. These findings establish that duodenal lipid sensing activates a gut–brain–brown fat axis to determine brown fat temperature, and thereby reveal a previously unappreciated pathway that regulates thermogenesis.

## Introduction

The energetic relevance of brown adipose tissue (BAT) thermogenesis in adult human physiology has recently found new support after the publication of three independent studies consistently demonstrating the presence of metabolically-active BAT in adult humans using tomography technologies [1]–[3]. BAT thermogenesis is modulated by environmental temperature and diet through sympathetic innervation [4], [5]. The release of noradrenaline at sympathetic neuronal terminals on BAT adipocytes activates uncoupling protein 1, which diverts protons from the oxidative cycle, resulting in the dissipation of energy through the production of heat. Diet-induced thermogenesis is a significant contributor to energy expenditure, acutely increasing metabolic rate by ∼25–40% in humans and rodents [6], [7], and accounting for approximately 15% of energy expenditure in lean individuals [8], [9]. However, the mechanisms linking nutrient availability and BAT thermogenic activity remain poorly characterized.

Intraduodenal administration of lipids inhibits food intake in both rodents and humans [10], [11]. CCK is rapidly released from endocrine cells of the small intestine upon intraluminal fat infusion [12]. Within the small intestinal mucosa, gut vagal afferent fibers are in close apposition to CCK immunoreactive endocrine cells [13], CCKA receptors are expressed on and transported in gut vagal afferents [14], [15], and CCKA receptors mediate the anorexigenic effect of intestinal lipids [11]. Surgical or chemical destruction of vagal afferents block the feeding inhibitory effects of duodenal lipids and intraperitoneal CCK [16]. The feeding inhibitory actions of peripheral CCK are also mediated by NMDA receptors in the caudomedial nucleus of the solitary tract (NTS), where gut vagal afferents terminate [17]. Finally, duodenal fat infusion also activates this gut-CCK -NTS glutamatergic pathway to limit nutrient availability by suppressing hepatic glucose production [18]. These data support a gut-brainstem axis critical in the negative feedback control of ingestion and glucose homeostasis following duodenal lipid infusions.

Interestingly, the NTS also innervates the BAT [19], regulates sympathetic tone to BAT [20] and has been directly implicated in the control of thermogenesis [21], [22]. However, a potential role for a gut-NTS-BAT axis in the control of thermogenesis in response to duodenal fat sensing remains unexplored. Consequently, the purpose of these experiments was to identify the degree to which the gut-brain axis could be implicated in the thermogenic effects of gut nutrient infusions.

## Materials and Methods

### Animals and Reagents

Sprague Dawley rats (275–300 g, Charles River Laboratories) were housed in individual cages with wire mesh bottoms and maintained in a temperature-controlled room under a standard 12 h/12 h light/dark cycle with ad libitum access to water and standard chow unless specifically indicated. Animals were handled daily and trained to be connected to the injection systems for the 5 days preceding the experiments. All experimental protocols were approved by the Institute for Animal Studies of the Albert Einstein College of Medicine.

### Drugs

Intralipid 20% (IL, VWR) was dissolved in saline (50∶50) to a 1 kcal/ml emulsion. The topical anesthetic tetracaine-HCl (Sigma) was dissolved to a 5% solution in saline and used at a final concentration of 360 µg/ml, consistent with doses used to block intestinal extrinsic neural afferent activity and the feeding inhibitory effects of duodenal lipid infusions [10]. The CCKA receptor antagonist devazepide (DVZ, Tocris) was prepared by dissolving 10 mg in 0.1 ml dimethyl sulfoxide (DMSO, Sigma), adding 0.1 ml Tween 80 (Sigma), followed by 0.8 ml physiological saline. This stock solution was diluted in physiological saline to achieve a final concentration of 1 mg/ml. DVZ was administered ip at a dose of 1 mg/kg, consistent with doses used to block feeding inhibitory effects of duodenal lipids and CCK [23]. The N-methyl-d-aspartate (NMDA) receptor antagonist MK-801 (Tocris) was prepared in sterile artificial cerebro-spinal fluid (aCSF, Tocris) to a final concentration of 1 µM.

### Animal Preparation

Rats were overnight food deprived before surgical procedures that were performed under ketamine/xylazine anesthesia. Rats underwent duodenal cannulation, as previously described [24], and infusion catheters were placed in the duodenum 2 cm from the pyloric sphincter. Rats were also implanted with a temperature transponder under the interscapular BAT (Mini-mitter, Philips Respironics). Following the duodenal catheter implantation, animals were maintained on nutritionally complete Ensure vanilla liquid diet (Abbott) and the duodenal catheter was flushed with 0.3–0.5 ml saline once daily to ensure patency.

An additional group of rats was also stereotaxically implanted with a bilateral steel guide cannula (Plastics One) positioned 2 mm above the caudomedial nucleus of the solitary tract (cannula holding bar in a 10° rostro-caudal angle, coordinates relative to occipital suture: A/P +1.5 mm, D/V −6.8 mm. +/−0.75 lateral to midline). Beveled stainless steel injectors (33 gauge) extending 2.0 mm from the tip of the guide cannulas were used for injections. Accurate cannula placement was confirmed by consumption of more than 1.5 g of chow within the 60 minutes following a parenchymal injection of 24 µg of 5-thio-D-glucose (Sigma) in 100 nl of aCSF per side [25]. All animals were allowed a 1 week surgical recovery period before the beginning of the experiments.

### Injection Protocols

All injections were performed in a crossover manner and at least 4 days elapsed between each injection. Rats were fasted overnight, attached to a freely moving dual channel swivel assembly (Instech) mounted on their home cages, 2 hours before the beginning of the injections. Home cages were located atop ER-4000 receivers (Philips Respironics), and BAT temperature was monitored during the 2 h preceding and the 2 h following the beginning of the duodenal infusion, in a temperature-controlled room maintained at 72°F. On four separate test days, rats received a 30 min intraduodenal (ID) infusion of one four infusates: 5 ml saline, 5 ml saline +1.8 mg tetracaine-HCl, 5 ml IL 10%, or 5 ml IL 10% +1.8 mg tetracaine-HCl. In the DVZ experiment, rats received an intraperitoneal (ip) injection of 350 µl of saline or 1 mg/kg DVZ 15 min prior to an ID infusion of 5 ml saline or IL 10%. In the MK801 experiments, rats received a 2 min bilateral NTS injection of 100 nl aCSF or MK801 (0.1 nmol/side) 15 min prior to an ID infusion of 5 ml saline or IL 10%.

### Statistical Analysis

All data, presented as means ± SEM, were analyzed using GraphPad Prism 5. For all statistical tests, an α risk of 5% was used. Data were analyzed using a mixed model ANOVA for repeated measurements and post-hoc tests were performed using Bonferroni correction.

## Results and Discussion

### Duodenal Lipid Sensing Increases BAT Temperature

**Figure 1:**
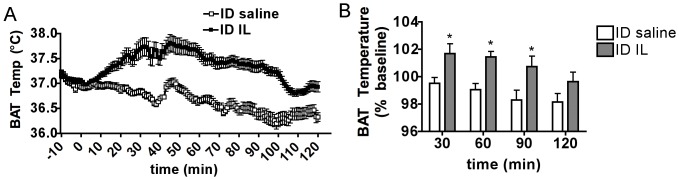
Duodenal lipid sensing increases BAT temperature. (A) BAT temperature 10 min prior to and 120 min following a 30 min intraduodenal infusion of 5 ml saline or IL 10%. (B) BAT temperature expressed as a % of baseline at 30, 60, 90 and 120 min following the beginning of a 30 min intraduodenal infusion of 5 ml saline or IL 10%. All data are means ± SEM, n = 7. *: p<0.05.

Intraduodenal saline infusion in overnight fasted rats minimally affected BAT temperature, which non-significantly decreased by 0.47±0.24°C during the 2 h following the beginning of the infusion (Figure 1). In contrast, intraduodenal IL induced a significant increase in BAT temperature beginning 20 min after the onset of the infusion and sustained throughout the following 80 min (Figure 1). The BAT temperature increment from baseline peaked at 1.27±0.18°C at 44.3±9.9 min following the beginning of the infusion. These results reveal for the first time that acute increases in duodenal nutrient availability induce changes in BAT thermogenic activity.

### Blockade of Intestinal Vagal Sensory Fibers Blunts Duodenal Lipid-induced BAT Thermogenesis

**Figure 2:**
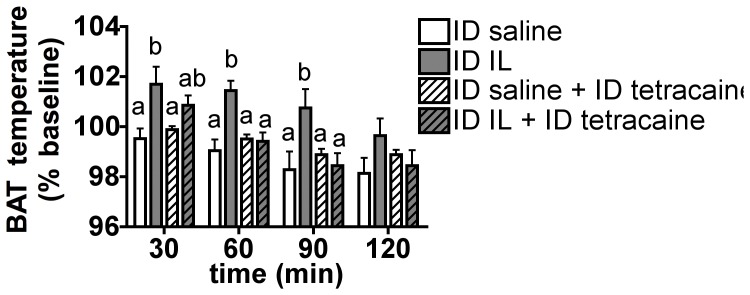
Pharmacological blockade of small intestinal sensorineural function blunts duodenal lipid-induced BAT thermogenesis. BAT temperature expressed as a % of baseline at 30, 60, 90 and 120 min following the beginning of a 30 min intraduodenal infusion of 5 ml saline or IL 10%, alone or together with 1.8 mg of tetracaine. All data are means ± SEM, n = 6–7. Means that share a common letter do not significantly differ from one another.

We then asked whether activation of intestinal sensory fibers was implicated in the thermogenic effect induced by duodenal lipids using the local anesthetic tetracaine. Duodenal tetracaine blocks the ability of nutrients to suppress food intake and hepatic glucose production, without having any effect on food intake or glucose homeostasis when administered alone [18] [10]. Intraduodenal tetracaine treatment during intraduodenal saline infusion had no effect on BAT temperature (Figure 2). When infused together with IL, tetracaine partially blunted the early increase in BAT temperature induced by IL alone 30 min after the beginning of the infusion, and completely blocked the IL-induced increase of BAT temperature during the remainder of the test (Figure 2). These results support the interpretation that activation of afferent nerve fibers innervating the upper intestine is required for the thermogenic effect of intraduodenal IL, and thus that lipids engage a neuronal network to regulate BAT activity.

### CCKA Receptor Blockade Blunts Duodenal Lipid-induced BAT Thermogenesis

**Figure 3:**
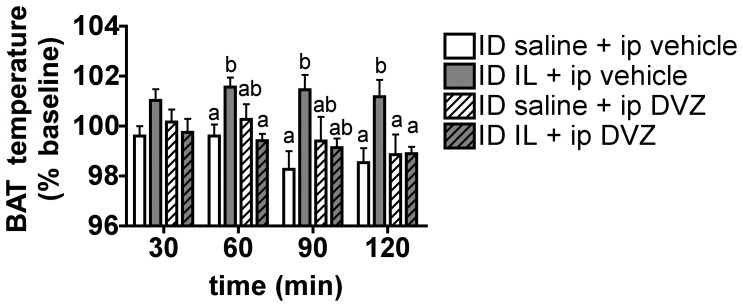
CCKA receptor blockade blunts duodenal lipid-induced BAT thermogenesis. BAT temperature expressed as a % of baseline at 30, 60, 90 and 120 min following the beginning of a 30 min intraduodenal infusion of 5 ml saline or IL 10% and preceded by an intraperitoneal injection of DVZ or vehicle. All data are means ± SEM, n = 6–7. Means that share a common letter do no significantly differ from one another.

Results from multiple studies support the idea that CCKA receptors on gut vagal afferents play critical roles in relaying duodenal lipid signals important in the negative feedback control food intake and glucose homeostasis [11] [17]–[18]. The present data suggest that the ability of tetracaine to block duodenal lipid induced thermogenesis is also due to blockade of gut vagal CCKA receptors. Thus, we tested whether activation of CCKA receptors is required for the thermogenic effect of duodenal lipids using the CCKA receptor blocker Devazepide (DVZ). Pre-treatment with DVZ did not affect BAT temperature following saline duodenal infusion, whereas DVZ prevented the intraduodenal IL-induced increase in BAT temperature, consistent with the idea that duodenal lipids activate CCK-A receptors on vagal sensory fibers to regulate BAT thermogenic activity (Figure 3).

### NTS NMDA Receptor Blockade Blunts Duodenal Lipid-induced BAT Thermogenesis

**Figure 4:**
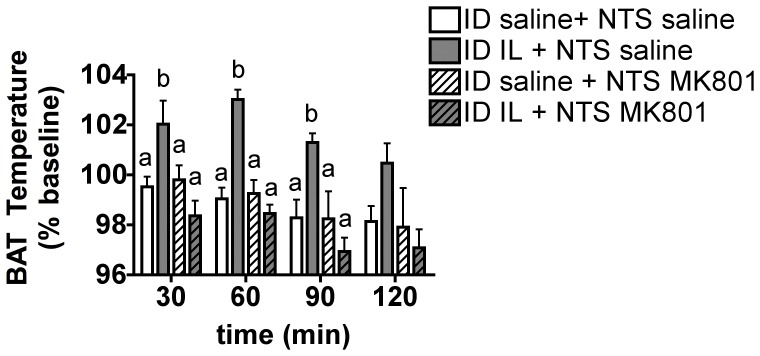
NTS NMDA receptor blockade blunts duodenal lipid-induced BAT thermogenesis. BAT temperature expressed as a % of baseline at 30, 60, 90 and 120 min following the beginning of a 30 min intraduodenal infusion of 5 ml saline or IL 10% and preceded with an bilateral NTS injection of MK801 or vehicle. All data are means ± SEM, n = 7. Means that share a common letter do no significantly differ from one another.

N-methyl-d-aspartate (NMDA) receptors have been localized to vagal afferent terminals in the hindbrain NTS [26], [27] and have been implicated in CCK-induced anorexia [17] and duodenal lipid-induced reduction of hepatic glucose production [18]. Thus, we tested their role in duodenal lipid-induced thermogenesis. Local bilateral NTS administration of the NMDA receptor blocker MK801 did not affect the BAT temperature response to intraduodenal saline (Figure 4). In contrast, NTS MK801 treatment prior to intraduodenal IL infusion completely suppressed the increase in BAT temperature induced by intraduodenal IL infusion (Figure 4). These results indicate that activation of NTS NMDA receptor signaling is required for duodenal lipid-induced thermogenesis and support the conclusion that NTS glutamatergic transmission relays afferent neuronal signals triggered by duodenal lipids to increase BAT thermogenesis.

Taken together, these data support the suggestion that duodenal lipid sensing activates a neurocircuit engaging CCKA receptors on vagal sensory fibers and their glutamatergic projections in the NTS to regulate BAT temperature, revealing a role for a gut-brain-BAT axis in the control of thermogenesis.

The present data are consistent with previous findings demonstrating that duodenal lipid infusions in awake behaving rats at doses much lower than those employed in the present study, delivered at comparable rates (4.5 vs 5 ml/30 min), elicit robust c-fos activation in NTS at the level of the AP [27], [28]. While brainstem c-fos is used an index of neuronal activation, it does not directly reflect the neuronal activity of the c-fos labeled cells at the time of the duodenal infusions. Neurophysiological recordings from the NTS, especially in awake animals, would provide additional compelling demonstrations of this.

We cannot rule out the possibility that the gut luminal exposure to tetracaine may non-specifically modulate local gut neural function in ways not related to the vagal afferent signaling of duodenal lipid availability. For example, as tetracaine is an anesthetic, it might be anticipated to affect intrinsic enteric gut neurons that determine local gut motility. Furthermore, local luminal distribution of lipid infusion may have been affected by tetracaine. However, doses two times greater than those used in the present study have been shown to induce minimal changes in duodenal, colonic or gastric motility, and have no effect on acetylcholine-induced contractions of the isolated rat duodenum, colon or stomach [30]. In addition, tetracaine at similar doses had no effect on the potential difference across duodenal segments for the first 45 minutes of exposure [31], during which lipid-induced BAT thermogenesis was most pronounced, and when duodenal tetracaine coadministration most completely blocked the BAT response. These data support the interpretation that the tetracaine used in the present study did not have a generalized effect on active transport across the external membrane of the duodenal mucosa. In contrast to these findings, tetracaine has clearly been shown to block electrical stimulation induced action potentials in vagal sensory fibers [32].

It is also important to consider that we do not directly demonstrate that duodenal tetracaine blocks the ability of lipid to activate the proposed NTS component of the gut-brainstem BAT circuit. For example, gut tetracaine would be predicted to reduce the NTS c-fos response to duodenal lipids under these circumstances. However, damage to the gut afferent vagus nerve has been shown to block the ability of duodenal lipid to stimulate NTS c-fos [33], and to inhibit feeding [34], and tetracaine does block afferent vagal action potentials [32]. Together, these findings support the interpretation that gut tetracaine interrupts gut vagal afferent lipid signals from reaching the NTS.

The present data do not directly address the degree to which CCKA receptors specifically within gut vagal afferents are responsible for the ability of duodenal lipids to increase BAT thermogenesis. Prior studies demonstrated that intestinal infusions of MK329 were sufficient to block the ability of duodenal CCK to inhibit hepatic glucose production [36], but these studies did not preclude the possibility that the MK329 acted outside the lumen, at other peripheral or central CCKA receptor sites. Furthermore, neither total nor selective vagal afferent transection procedures would directly address the specific roles for small intestinal vagal afferent integrity in the present effects, as both of these procedures inevitably involve additional interruption of vagal afferent signals from gut sites outside the small intestine. However, the fact that both duodenal administration of tetracaine and systemic devazepide were able to block duodenal lipid induced thermogenesis argues for an important role of gut vagal afferent CCKA receptors in this circuit.

Based on the ability of the CCKA receptor antagonist devazepide to block duodenal lipid induced temperature elevations, it is reasonable to anticipate that other nutrient secretagogues of CCK, such as proteins and amino acids, would also stimulate BAT thermogenesis via this pathway. Conversely, nutrients that are poor plasma CCK secretagogues, such as carbohydrates [35] would not be predicted to be effective stimuli.

Vagal afferent fibers innervating the proximal small intestine, as well as their brainstem terminations at the caudomedial level of the nucleus of the solitary tract, express both NR1 and NR2 NMDA receptor subunits [37], [38]. Furthermore, pharmacological inhibition of NR2 NMDA receptor subunits within the caudal brainstem blocks the feeding inhibitory actions of CCK and the ability of duodenal CCK to reduce hepatic glucose production [39], [40]. The present data demonstrating that parenchymal NTS MK801 injection blocks the ability of duodenal lipid infusions to drive BAT thermogenesis suggests that brainstem NR2 NMDA receptor subunits on gut vagal afferent terminations or their recipient NTS neurons mediate this effect.

The present effects of NMDA receptor blockade may not be not strictly limited to the stereotaxically targeted medial NTS through the rostrocaudal extent of the are postrema (AP). However, we have recently demonstrated that labeled tracer injections targeting the mNTS, delivered at equivalent rates and volumes (100 nl/2 min), were confined to the medial NTS at the level of the AP, and did not extend into the subjacent dorsal motor vagal nucleus (DMX) at this level, nor do they impinge on the DMX at more rostral or caudal levels of the brainstem [29].

The molecular and neuroanatomical pathways linking NTS glutamatergic stimulation to BAT thermogenesis remain unclear, but GABAergic modulation of brainstem glutamatergic transmission may be important. Disinhibition of medial NTS neuronal activity using GABA antagonists blocks the BAT thermogenic response to cold challenges, and NTS GABA agonist administration facilitates BAT thermogenesis via sympathetic premotor neurons in the raphe pallidus [21]. GABAA receptors have been localized to vagal afferent glutamatergic terminations within the NTS, and GABAA receptors mediate the release of glutamate from vagal afferents [41], [42]. Taken together these data suggest that GABAA signaling on glutamatergic gut vagal afferents may modulate postsynaptic input to NTS neurons critical in BAT thermogenesis.

In summary, the present data identify a novel role for gut nutrient sensing in the control of BAT thermogenesis, a potentially important contributor to overall energy balance. This gut-brain-BAT circuit identifies multiple peripheral and central neuroendocrine nodes where pharmacological or nutrient intervention may reveal new molecular therapeutic targets that modulate energy expenditure in obesity and related metabolic diseases.
